# Level of mitoses in non-muscle invasive papillary urothelial carcinomas (pTa and pT1) at initial bladder biopsy is a simple and powerful predictor of clinical outcome: a multi-center study in South Korea

**DOI:** 10.1186/s13000-017-0639-y

**Published:** 2017-07-24

**Authors:** Ji Eun Kwon, Nam Hoon Cho, Yeong-Jin Choi, So Dug Lim, Yong Mee Cho, Sun Young Jun, Sanghui Park, Young A. Kim, Sung-Sun Kim, Mi Sun Choe, Jung-dong Lee, Dae Yong Kang, Jae Y. Ro, Hyun-Jung Kim

**Affiliations:** 1 Department of Pathology, Inje University Sanggye Paik Hospital, 1342, Dongilro, Nowon-gu, Seoul, South Korea; 2 Department of Pathology, Ajou University school of Medicine, Suwon, South Korea; 3 Department of Pathology, Yonsei Medical College of Medicine, Seoul, South Korea; 4 Department of Pathology, Seoul St Mary’s Hospital, The Catholic University, Seoul, South Korea; 5 Department of Pathology, Konkuk University Medical center, Konkuk University School of Medicine, Seoul, South Korea; 6 Department of Pathology, Asan Medical Center, Ulsan College of Medicine, Seoul, South Korea; 7 Department of Pathology, Inchun St. Mary’s Hospital, The Catholic University, Incheon, South Korea; 8 Department of Pathology, College of Medicine, Ewha Womens University, Seoul, South Korea; 9 Department of Pathology, SMG-SNU Boramae Medical Center, Seoul, South Korea; 10 Departments of Pathology, Chonnam National University Medical school, Gwangju, South Korea; 11 Department of Pathology, Keimyung University School of Medicine, Daegu, South Korea; 12 Office of Biostatistics, Ajou University, School of Medicine, Suwon, South Korea; 13 Department of Pathology, Houston Methodist Hospital, Weill Medical College of Cornell University, New York, USA

**Keywords:** Predictor, Clinical outcome, Mitotic level, Papillary urothelial carcinoma, Predictor

## Abstract

**Background:**

Histologic grade is the most important predictor of the clinical outcome of non-muscle invasive (Ta, T1) papillary urothelial carcinoma (NMIPUCa), but its ambiguous criteria diminish its power to predict recurrence/progression for individual patients. We attempted to find an objective and reproducible histologic predictor of NMIPUCa that correlates well with the clinical outcome.

**Methods:**

A total of 296 PUCas were collected from the Departments of Surgical Pathology of 11 institutions in South Korea. The clinical outcome was grouped into no event (NE), recurrence (R), and progression (P) categories. All 25 histological parameters were numerically redefined. The clinical pathology of each case was reviewed individually by 11 pathologists from 11 institutions based on the 2004 WHO criteria and afterwards blindly evaluated by two participants, based on our proposed parameters. Univariate and multivariate logistic regression analyses were performed using the R software package.

**Results:**

The level of mitoses was the most reliable parameter for predicting the clinical outcome. We propose a four-tiered grading system based on mitotic count (> 10/10 high-power fields), nuclear pleomorphism (smallest-to-largest ratio of tumor nuclei >20), presence of divergent histology, and capillary proliferation (> 20 capillary lumina per papillary core).

**Conclusions:**

The level of mitoses at the initial bladder biopsy and transurethral resection (TUR) specimen appeared to be an independent predictor of the Ta PUCa outcome. Other parameters include the number of mitoses, nuclear pleomorphism, divergent histology, and capillary proliferation within the fibrovascular core. These findings may improve selection of patients for a therapeutic strategy as compared to previous grading systems.

**Electronic supplementary material:**

The online version of this article (doi:10.1186/s13000-017-0639-y) contains supplementary material, which is available to authorized users.

## Background

Non-muscle invasive papillary urothelial carcinomas (NMIPUC) of the urinary bladder, with tumors staged as non-invasive intraepithelial (Ta) or tumors with invasion of the lamina propria/submucosa (T1), are known to recur frequently (in up to 70% of cases), and occasionally (in up to 40% of cases) progress [1]. The European Association of Urology (EAU) guidelines for NMIPUC-(pTa/T1) of the bladder proposed risk stratification for progression into low-, intermediate-, and high-risk groups [2]. The same guideline stated that the patients in different groups should be managed using different strategies. Besides stage (Ta vs. T1), size (< 3 cm vs. > 3 cm), number of papillary tumors (single vs. multiple), concurrent carcinoma in situ (CIS), and a history of recurrence, the best estimator of risk is the histological grade. The four existing grading systems (1973 World Health Organization [WHO], 1998 International Society of Urologic Pathology [ISUP]/2004 WHO, Cheng et al. [3, 4], and 2016 WHO classifications) have divided PUCs based on subjective morphological parameters, which has led to a high interobserver/intraobserver variability in diagnoses made by pathologists, as well as lower predictive power in management by urologists [5]. In view of this, alternative grading systems have been sought to improve the grading discrepancy [6]. Many studies on immunohistochemical and molecular markers have been conducted to reduce the subjectivity of the histological grading systems, but the markers studied have been declared as having no potential for playing a role in grading schemes [7–12].

The present study was conducted to identify more objective and reproducible histological predictors that may correlate well with the clinical outcome, and compare these to the previous histological grading systems. Eleven uropathologists evaluated light microscopic histological parameters together in three rounds, using a multihead microscope. Through this study, vigorous attempts were made to select all possible histological parameters as countable variables. Each parameter was evaluated using univariate and multivariate analyses, to determine whether these variables had statistically significant effect in predicting the clinical outcome.

## Methods

### Patient selection

Surgically removed NMIPUCs of the urinary bladder were collected from the surgical pathology archives of 11 institutions in South Korea. The inclusion criteria were as follows: (1) pTa or pT1 stage at the initial bladder biopsy; and (2) a 5-year minimum follow-up period for non-event (NE) cases. The exclusion criteria, on the other hand, were as follows: (1) a prior history or the concurrent presence of urothelial carcinoma either in the ureter or in the renal pelvis; and (2) evidence of associated urothelial carcinoma in situ. A total of 296 cases were retrieved (Ta, 178; T1, 118). The number of cases contributed by each institute was 95, 47, 38, 22, 21, 21, 17, 15, 14, 4, and 2.

### Clinical parameters

The retrieved cases were classified into three clinical subgroups: no event (NE), recurrence (R), and progression (P). NE was defined as cases with no evidence of tumor on the follow-up imaging study, urine cytology, or cystoscopy for at least 5-year follow-up duration; R was defined as cases showing a new tumor occurrence with the same or lower stage at least 3 months after the initial resection; and P was defined as a cases showing new tumor development with a higher stage than the initial stage, or metastasis to the lymph nodes or other organs. We collected clinical information on the patients from the medical records, including 1) age, 2) sex, 3) site, number, and size/volume of tumor in first biopsy, 4) interval to the 2nd event, 5) number of recurrences, 6) the type of final operation, 7) survival, 8) cause of death, and 9) site of metastasis. All types of specimens (cystoscopic biopsy, cold-cup biopsy, transurethral resection of bladder tumor) were included, but were not defined separately. However, there was no partial or radical cystectomy specimen (as an initial biopsy) among the 296 cases. The number of tumors was divided into two groups: single vs. multiple. The tumor size was also divided into two groups: < 3 cm vs. > 3 cm. The distribution of each group is shown in Table 1.

**Table 1:** Clinical characteristics of the patients

	Stage	
	Ta (*n* = 178)	T1 (*n* = 118)
Clinical subgroup (NE/R/P)	73/69/37	32/50/30
Age (mean)	65	68
Sex (M/F)	138/40	93/25
Number of tumor (single vs. multiple)	99/79	46/70^a^
Size (≤3 cm vs. > 3 cm)	130/48	68/50

*NE* No event, *R* Recurrence, *P* Progression

^a^not available in 2 cases

### Histological evaluation

**Fig. 1:**
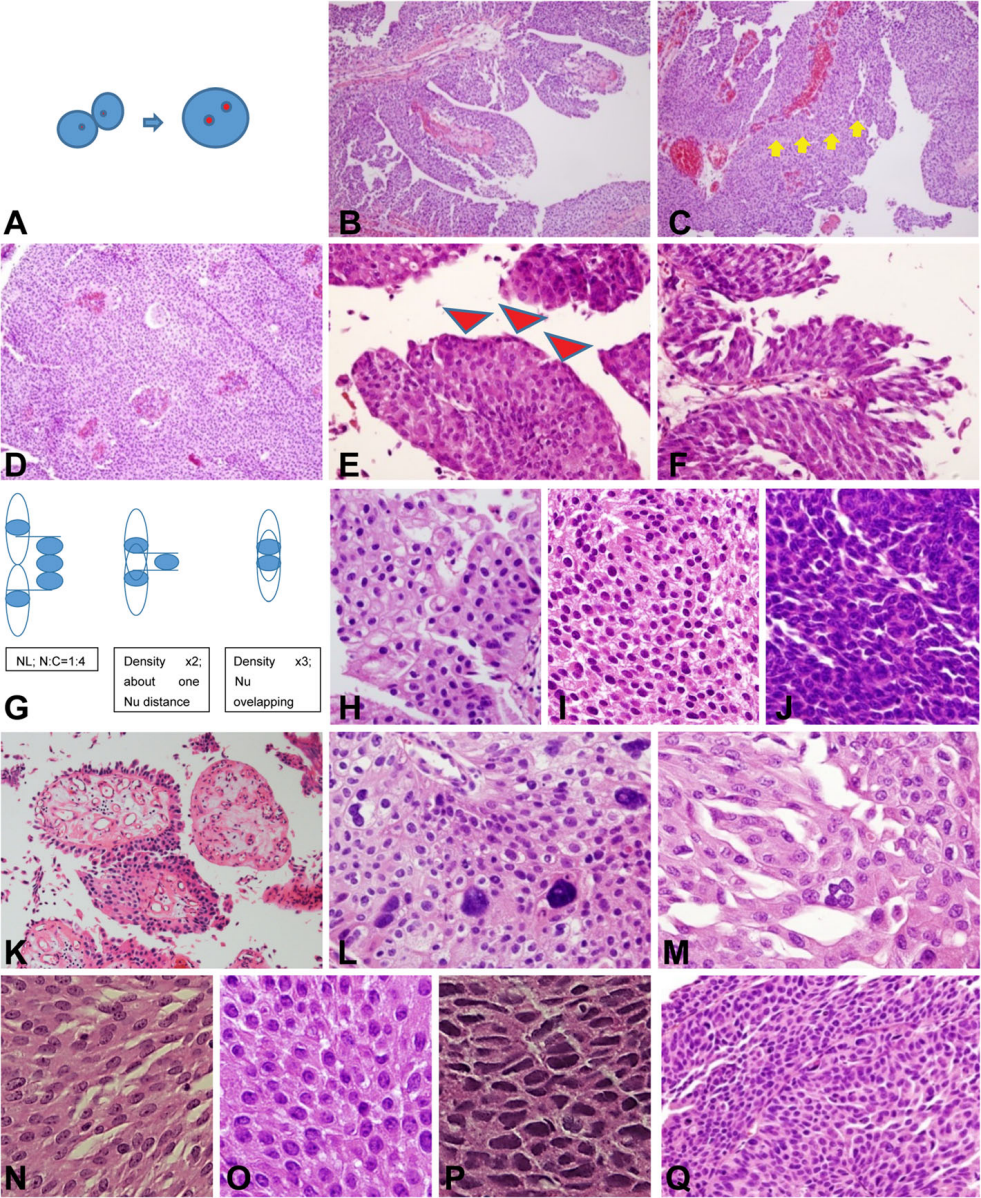
(1) Representative images of histological parameters evaluated in this study. **a** Schematic figure of papillary fusion. **b** Delicate papillae with no fusion. **c** Papillary fusion (the arrow marks an imaginary fusion line). **d** Confluent fusion of papillae. **e** Presence of umbrella cells (arrowhead). **f** Absence of umbrella cells. **g** Schematic figure for the estimation of cell density based on the distance between cells. **h** Cell density score 1. **i** Cell density score 2. **j** Cell density score 3. **k** Discohesiveness (**l**). Nuclear pleomorphism category 4 based on a difference between the smallest and the biggest nucleus of the tumor cells of about 20-fold. **m** Multinucleated giant cells. **n** Mild nuclear hyperchromasia. **o** Moderate nuclear hyperchromasia. **p** Severe nuclear hyperchromasia. **q** Polarity loss score 2

**Fig. 2:**
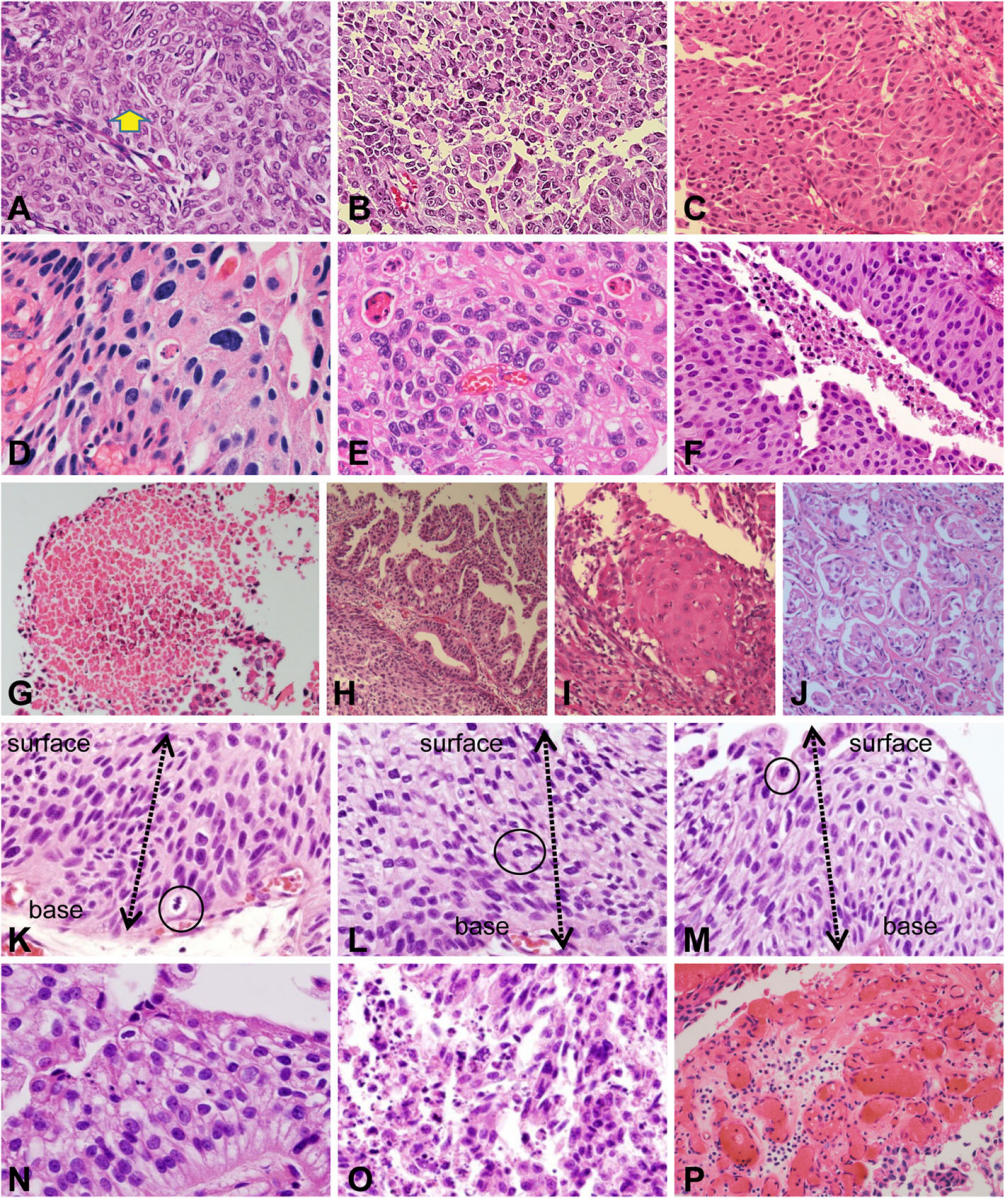
(2) Further representative images of histological parameters evaluated in this study. **a** Example of a nuclear groove (arrow). **b** Prominent nucleoli. **c** Whorling pattern. **d** Single spotty necrosis (arrow). **e** Multifocal group necrosis (arrows). **f** Surface necrosis. **g** Confluent necrosis. **h** Glandular differentiation. **i** Squamous differentiation. **j** Micropapillary differentiation. **k** Mitosis level 1. **l** Mitosis level 2. **m** Mitosis level 3. **n** Apoptosis score 1. **o** Apoptosis score 3. **p** Capillary proliferation in fibrovascular core of papilla

For microscopic examination, hematoxylin and eosin (H&E)-stained glass slides of formalin-fixed, paraffin-embedded tissue of the tumors were retrieved. Interobserver discrepancy had been solved through several round-table multihead microscopic examinations involving 11 pathologists from 11 institutions, during which consensus opinion was reached. Although the proper muscle inclusion was not verified by the reviewers in all samples, 11 contributors had reviewed the original diagnosis and pathological stage, not only by a slide review, but also from the surgical records. The 2nd biopsy was routinely performed 3 months later for check-up of incomplete resection (i.e., residual tumor). Even if the initial diagnosis was NMIPUCa (Ta, T1), the cases with a short-interval change in the T stage were excluded and were regarded as an inaccurate diagnosis. The clinical pathology of each case was reviewed individually by 11 pathologists from 11 institutions based on the 2004 WHO criteria and was afterward blindly evaluated by two participants, based on our proposed parameters. The histological parameters that were examined are shown in Table 2 and Figs. 1 and 2. For prediction comparison, the previous grading systems, i.e., the 2004 WHO grading, Papillary urothelial neoplasm low malignant potential (PUNLMP) /Low grade (LG) /High grade (HG), 1973 WHO, Transitional cell carcinoma (TCC) grade 1/2/3, and Cheng et al., grade 1/2/3/4, were utilized [3, 4].

**Table 2:** Histologic parameters evaluated in this study

Variables	Definition	Category	Explanation of category
Papillary fusion	Fusion of papillae with forming confluent and complex papillary cores	1	<1/3 area
		2	1/3 ~ 2/3 area
		3	>2/3 area
Umbrella cells	Area with preserved umbrella cells	1	>50% area
		2	5-50% area
		3	<5% area
Discohesiveness	Detached cells from papillae	1	<1/3 area
	Refer to Additional file 1: Figure S1.	2	1/3 ~ 2/3 area
		3	>2/3 area
Cell density		1	<1/3 areas show more than 2 times normal density
		2	1/3 ~ ≤2/3 area shows more than 2 times of normal density
		3	>2/3 area show more than 2 times normal density or >5% area shows more than 3 times normal density
Nuclear pleomorphism	Size of smallest nuclei vs. largest nuclei(regardless of tangential sectioning)	1	<3
		2	3 ≤ and <8
		3	8 ≤ and <20
		4	≥20
Multinucleated giant cells	Presence of bi-or multinucleated nuclei	0	Absence
		1	Presence
Loss of polarity	Proportion of cells deviating from the vertical alignment shown in normal urothelium	1	<5%
		2	5-50%
		3	>50%
Hyperchromasia	Semiquantitative degree of nuclear hyperchromasia	1	diffusely mild
		2	diffusely moderate or focally strong
		3	diffusely strong
Nuclear groove	Proportion of tumor cells without identifiable nuclear grooves	1	<5%
		2	5-50%
		3	> 50%
Prominent nucleoli	Proportion of cells having prominent nuclei (recognizable under 10× medium power)	1	<5%
		2	5-50%
		3	>50%
Whorling pattern	Refer to Additional file 1: Figure S1.	0	absent
		1	present
Necrosis	Degree of necrosis	1	singly spotted
		2	focally grouped or multifocally spotted
		3	surface necrosis of nests
		4	confluent necrosis
Divergent histology	Presence and number of glandular, squamous or micropapillary differentiation	0	absence
		1	1
		2	2
		3	3
Mitotic count	Number of mitosis/ 10 consecutive HPFs in most mitotically active area	CON	
Mitotic count-CAT	Group of mitotic count	1	0-2
		2	3-7
		3	8-15
		4	≥16
Mitotic level	Most highest level of mitotic figures, from base to the top of the papilla(low 1/3, mid1/3, and high 1/3)	1	<1/3 or no mitosis
		2	1/3-2/3
		3	>2/3
Apoptosis	Number of apoptotic bodies in most active area/one HPF	1	<10
		2	10-100
		3	>100
Capillary proliferation	Number of capillary in one papillary core	CON	

*CAT* Categorical variable, *CON* Continuous variable, *HPF(s)* High power field(s)

### Statistical analysis

All of the aforementioned parameters were evaluated in two paired comparison groups (i.e., R vs. NE and P vs. NE) at each stage. To identify the factors influencing R and/or P, univariate and multivariate logistic regression analyses were performed. To investigate the diagnostic utility of the new grading system, it was compared with the previous grading systems by area under the curve (AUC) of receiver operating characteristics (ROC) curves. All the statistical analyses were performed in the R software package (R version 3.1.2, R Foundation for Statistical Computing, Vienna, Austria; <http://www.R-project.org/ >).

## Results

### Univariate analysis

For PUC-Ta, among morphologic variables, the number (odds ratio [OR] 0.34 [95% confidence interval, CI: 0.17-0.67]; *p*-value = 0.002), size (OR 2.27 [95% CI: 1.05-5.07]; *p*-value = 0.0399), mitotic count (OR 1.03 [95% CI: 1.00-1.07]; *p*-value = 0.0468), mitotic level (OR 1.09 [95% CI: 0.24-4.83]; *p*-value = 0.010), and capillary proliferation in fibrovascular cores (OR 1.05 [95% CI: 1.01-1.10]; *p*-value = 0.0136) were associated with tumor recurrence. Nuclear pleomorphism showed borderline significance for association with recurrence of PUC-Ta (Table 3). The factors associated with PUC-Ta progression included patient age, cell density, nuclear pleomorphism, hyperchromasia, nuclear groove, prominent nucleoli, necrosis, mitotic count, mitotic level. Capillary proliferation and apoptosis had borderline statistical significance (Table 4). For PUC-T1, the whorling pattern was associated with recurrence and the mitotic level showed borderline significant association with recurrence. Divergent histology was associated with progression only (Additional file 1: Tables S1 and S2).

**Table 3:** Univariate analysis of factors associated with recurrence of PUC-Ta

Variable	Category	Odds ratio	95% Confidence Interval	*P*-value
Age		1.00	0.97-1.03	0.9401
Sex	man vs. woman	1.11	0.50-2.47	0.7908
No of tumor		0.34	0.17-0.67	0.0021*
Size of tumor		2.27	1.05-5.07	0.0399*
Papillary fusion	2 vs 1	1.50	0.65-3.50	0.3430
	3 vs 1	1.60	0.74-3.50	0.2348
Umbrella cell	2 vs. 1	0.95	0.44-2.07	0.9020
	3 vs. 1	1.19	0.52-2.75	0.6723
Discohesiveness	2 vs. 1	1.93	0.80-4.78	0.1480
	3 vs. 1	1.57	0.71-3.53	0.2682
Cell density	2 vs. 1	1.62	0.69-3.88	0.2737
	3 vs. 1	0.88	0.37-2.08	0.7719
Nuclear pleomorphism	2 vs. 1	0.47	0.21-1.03	0.0621
	3 vs. 1	0.39	0.14-1.03	0.0599
	4 vs. 1	3.84	0.58-76.11	0.2324
Multinucleated giant cell	1 vs. 0	1.14	0.58-2.24	0.7041
Loss of polarity	2 vs. 1	1.03	0.41-2.59	0.9557
	3 vs. 1	0.82	0.29-2.26	0.6981
Hyperchromasia	2 vs. 1	1.36	0.67-2.79	0.3904
	3 vs. 1	0.97	0.26-3.47	0.9604
Nuclear groove	2 vs. 1	1.83	0.86-3.96	0.1184
	3 vs. 1	1.60	0.65-3.99	0.3079
Prominent nucleoli	2 vs. 1	1.12	0.56-2.23	0.7491
	3 vs. 1	0.81	0.15-3.90	0.7903
Whorling pattern	1 vs. 0	1.07	0.55-2.11	0.8327
Necrosis	2 vs 1	0.98	0.39-2.48	0.9694
	3 vs 1	0.77	0.21-2.58	0.6680
	4 vs 1	1.47	0.54-4.14	0.4490
Divergent histology		2.04	0.73-8.10	0.2172
Mitotic count		1.03	1.00-1.07	0.0468*
Mitotic count (CAT)	2 vs. 1	4.62	1.89-11.98	0.0011
	3 vs. 1	2.52	0.94-6.95	0.0684
	4 vs. 1	3.15	1.22-8.46	0.0193*
Mitotic level	2 vs. 1	2.13	0.83-5.70	0.1229
	3 vs. 1	4.44	1.88-11.18	0.0010*
Apoptosis	2 vs 1	1.15	0.53-2.50	0.7246
	3 vs 1	1.09	0.24-4.82	0.9116
Capillary proliferation		1.05	1.01-1.10	0.0136*

*No* Number, *CAT* Categorical variable; *P < 0.05

**Table 4:** Univariate analysis of factors associated with progression of PUC- Ta

Variable	Category	Odds ratio	95% Confidence Interval	*P*-value
Age		1.05	1.01-1.09	0.0095*
Sex	man vs. woman	1.06	0.46-2.7	0.8893
No of tumor		0.61	0.29-1.27	0.1856
Size of tumor		1.40	0.62-3.03	0.4012
Papillary fusion	2 vs 1	1.27	0.52-3.04	0.5871
	3 vs 1	0.89	0.36-2.13	0.8044
Umbrella cell	2 vs. 1	1.82	0.64-5.13	0.2590
	3 vs. 1	2.71	0.96-7.62	0.0591
Discohesiveness	2 vs. 1	1.30	0.49-3.26	0.5841
	3 vs. 1	1.38	0.58-3.2	0.4579
Cell density	2 vs. 1	2.02	0.56-9.58	0.3165
	3 vs. 1	5.40	1.73-23.89	0.0093*
Nuclear pleomorphism	2 vs. 1	0.72	0.26-2.06	0.5331
	3 vs. 1	3.18	1.25-8.68	0.0180*
	4 vs. 1	1.46	0.19-7.53	0.6683
Multinucleated giant cell	1 vs. 0	1.56	0.75-3.24	0.2337
Loss of polarity	2 vs. 1	1.17	0.35-3.86	0.798
	3 vs. 1	2.70	0.82-8.93	0.104;
Hyperchromasia	2 vs. 1	2.42	1.16-5.2	0.0200*
	3 vs. 1	1.75	0.71-4.32	0.2206
Nuclear groove	2 vs. 1	9.26	3.44-26.31	0.0000*
	3 vs. 1	5.28	1.68-23.39	0.0105*
Prominent nucleoli	2 vs. 1	0.00	NA	0.9889
	3 vs. 1	0.37	0.14-0.87	0.0314*
Whorling pattern	1 vs. 0	1.25	0.26-4.87	0.7550
Necrosis	2 vs 1	2.04	0.70-5.59	0.1747
	3 vs 1	5.58	2.00-15.73	0.0010*
	4 vs 1	2.47	0.83-6.90	0.0903
Divergent histology		1.70	0.68-3.98	0.2222
Mitotic count		1.06	1.03-1.09	0.0000*
Mitotic count(CAT)	2 vs. 1	8.72	2.1-59.47	0.0076*
	3 vs. 1	8.45	1.8-60.71	0.0124*
	4 vs. 1	24.80	6.59-162.78	0.0000*
Mitotic level	2 vs. 1	7.20	1.23-136.92	0.0689
	3 vs. 1	15.51	3.1-282.16	0.0083*
Apoptosis	2 vs 1	2.18	0.97-4.84	0.0559
	3 vs 1	3.40	0.94-11.44	0.0501*
Capillary proliferation		1.03	1-1.07	0.0673

*NA* Not available, *CAT* Categorical variable; *P < 0.05

### Proposal of new grading system using more objective and fewer histological variables for predicting clinical outcome

Based on the univariate analysis results, three grades were designed for prediction of the biological behavior of PUC-Ta. The univariate analysis results for PUC-T1 revealed that only tumor stage influenced the biological behavior. Thus, once the tumor had invaded the lamina propria/submucosa, the histological parameters had an insignificant impact on the clinical outcome. Therefore, our new grading system was designed focusing on the prediction of PUC-Ta tumors. To design a new grading system with more objective and reproducible, yet simpler parameters, we chose mitotic level, mitotic count, capillary proliferation, and nuclear pleomorphism as important histological parameters, based on the univariate analysis. All four of these parameters not only had a statistically significant influence on both recurrence and progression of PUC-Ta, but were also quantifiable. Additionally, divergent histology was also selected as one of the parameters in our grading system; even though it showed an insignificant *p*-value in both recurrence and progression of PUC-Ta, it was statistically significant in terms of progression in PUC-T1 tumors.

Because the mitotic level appeared to be the most important morphological parameter based on the univariate analysis, the mitotic level was set as the first step in our proposed new grading system. Grades 1, 2, and 3 were assigned based on mitotic level, i.e., level 1, level 2, and level 3, respectively. In cases with any additional unfavorable histological features, including increased mitotic count (> 10/10 high-power fields), significant nuclear pleomorphism (smallest-to largest-ratio of tumor nuclei of >20), presence of divergent histology, and significant capillary proliferation (> 20 capillary lumina per papillary core), the tumors were upgraded: for example, grade 1 became grade 2, grade 2 became grade 3, and grade 3 became grade 4. We designed three similar but slightly different grading schemes.

### Comparison of our proposed grading system with previous grading systems

To investigate the diagnostic and prognostic utility of our proposed grading system, we compared the previous grading systems by comparison of AUC values in each system. All the statistical analyses were performed with adjustments for age, gender, tumor size, and number of tumors, to exclude the impact of factors other than histological parameters. For the prediction of recurrence of PUC-Ta, the AUCs of three previous grading systems were less than 0.7, whereas the AUCs of our proposed grading systems were over 0.7, and it was statistically significant (*p*-value <0.05). However, the differences between them were not statistically significant (Table 5). As for the prediction of progression of PUC-Ta, the AUCs of all of the previous and new grading systems were all larger than 0.7 (*p*-value <0.05) (Additional file 1: Figure S1).

**Table 5:** Comparison of AUC for predicting PUC-Ta tumor recurrence between previous grading systems and our proposed grading system

Old_grades	AUC-Old_grades (se)	Proposed_grade	AUC-Proposed_grade	Old vs. new grade *p*-value
#1	0.686 (0.044)	1	0.709 (0.043)	0.3188
		2	0.715 (0.043)	0.2784
		3	0.703 (0.044)	0.4289
#2	0.688 (0.045)	1	0.709 (0.043)	0.3800
		2	0.715 (0.043)	0.3215
		3	0.703 (0.044)	0.5003
#3	0.685 (0.045)	1	0.709 (0.043)	0.2809
		2	0.715 (0.043)	0.2516
		3	0.703 (0.044)	0.3599

#1:2004 WHO grading system (low grade/high grade); #2: 1973 WHO grading system (TCC grade 1,2, and 3); #3:Cheng et al. ‘s grading system [G1/G2/G3/G4-Anaplastic]

## Discussion

In this study, we attempted to find an objective and reproducible histologic predictor of NMIPUCa that correlates well with the clinical outcome and to compare these to the previous histological grading systems. We found that the level of mitoses at the initial bladder biopsy was an independent predictor of the Ta PUCa outcome; the number of mitoses, nuclear pleomorphism, divergent histology, and capillary proliferation within the fibrovascular core were also significant factors.

The EAU guideline proposed a three-risk group stratification. In addition to the tumor stage, tumor size, number of tumors, and association with CIS, histological grade was an important parameter for predicting progression [2, 5]. The 2004 and 2016 WHO grading systems had been modified from 1973 WHO classification; recently, in 2012, Cheng et al. developed a modified system. These systems are similar, but show slight variation. Each parameter was measured without well-defined criteria and has led to suboptimal reproducibility [13–15]. Each parameter was rated in terms of severity (mild/moderate/severe) or frequency (rare/occasionally/frequently). In routine pathology practice, pathologists often encounter a PUC of the bladder showing high mitotic activity, but only mild nuclear atypia and minimal loss of polarity, or in contrast, a case showing moderate nuclear pleomorphism and mild to moderate loss of polarity, but without discernible mitotic activity. In those cases, grading was not straightforward, because there was no priority finding depending on the weighted value among the many criteria, which complicated the grading assignment, and resulted in low reproducibility.

We attempted to develop a simple and reproducible grading system that could predict the clinical outcome in NMIPUC of the bladder. In this study, we included only cases with available initial-biopsy specimens and cases with no concurrent CIS. Initially all 11 uropathologists evaluated all histologic parameters using individual light microscopes, for three rounds. Twenty-five histological features with their numerical parameters (e.g., categorized grade or absolute number), including mitotic level and number of mitoses, level of apoptosis, necrosis, whirling appearance, and capillary proliferation, which had not been evaluated prior to this study, were selected, as well as other histological factors mentioned in the literature. Thereafter, two pathologists blindly evaluated all 296 cases to determine interobserver reproducibility. Some parameters appeared to be influenced by fixation and stain conditions. Therefore, intranuclear groove and nucleolar prominence, which may be produced by procedural artifacts, were considered as low-priority parameters.

In the univariate analysis of T1-stage tumors, only a divergent histology correlated with progression. We considered that the pathological stage-factor, with the presence of stromal (lamina propria/submucosal) invasion, was the most important factor dictating biological behavior from among the histological factors. This finding was in accordance with the WHO recommendation that grading is performed only for noninvasive PUC (PUC-Ta), and with other reports in the literature [16]. Therefore, in this study, the construction of the histological predictive model was limited to noninvasive (Ta) tumors, with exclusion of T1 tumors.

Unlike T1 tumors, Ta tumors had many clinical and histologic parameters that influenced the clinical outcome. Among the clinical factors, the number and size of tumors correlated with recurrence, while patient’s age was associated with progression. In terms of histological factors, mitotic count, mitotic level, and capillary proliferation correlated with recurrence. Cell density, nuclear pleomorphism, hyperchromasia, nuclear groove, prominent nucleoli, necrosis as well as mitotic count and level correlated with progression. Apoptosis and capillary proliferation disclosed borderline significance for progression.

It is worth noting that mitotic count showed the highest OR in the prediction of both recurrence and progression of PUC-Ta. In the early twenty-first century, many studies had focused on mitotic index (Ki-67, AgNO3) of Ta/T1 urothelial carcinomas, and have reported those as associated with tumor recurrence [17–19]. However, the impact of mitosis has not been fully evaluated for use, or has not been applied with a detailed cutoff-value in the grading system, in contrast to other epithelial cancers in other organs (low vs. high serous carcinoma of the ovary, histological grade of breast cancer and etc.) [20, 21]. Our results indicated that mitotic count should be integrated in the histological grading of PUC.

The importance of mitotic count has previously been emphasized for histological grading of NMIPUCa [22, 23]. Pich et al. showed that a high proliferative index is the most important recurrence-predictor among LMP and low-grade tumors [24]. Akkalp et al. also emphasized that higher mitotic activity (> 5/single high-power field) is a strong predictor for recurrence in Ta PUCa [25]. The studies indicate that proliferative activity can play an adjunctive role in histologic grading (even in low grade tumors) and prediction of recurrence or invasiveness, as also shown in this study. However, the criteria for proliferative activity were variable, including a mitotic count per one or 10 high power fields in any level of the neoplastic epithelium, and cut-off values for AgNOR and Ki-67. Considering that urothelial neoplasms are bulky, mitotic counting in high-power fields might be inconsistent and discordant.

Mitotic level has not received much attention either. The upper level of mitosis (level 3 mitosis) correlated with increased mitotic count and worse clinical outcome in our cohort. If a bulky mass is evaluated for the level of mitosis, the mitotic-specific marker, phospohistamine H3 (PHH3), can be useful for rapid detection of the mitotic level. PHH3 has been used for grading of upper-tract urothelial carcinoma [26]. Since the mitotic level and count were measurable, reproducible, and the most statistically significant parameters in our univariate analysis, we strongly recommended that these factors should be included as essential parameters in histological grading of PUC, even though identifying mitoses in an entire specimen requires marked effort.

HG tumors in the WHO 2004 and 2016 classification cover wide ranges of tumors from immediately above low-grade to highly anaplastic tumors. Recently, Cheng and his colleges suggested a four-tiered grading system that included grade 4, which consisted of an anaplastic group, and separating this group from the usual HG [3, 4, 27]. Because we agreed with the assignment of such an upper grade, the second step of our newly proposed grading scheme was focused on the selection of a more aggressive group. Four additional histological parameters (mitotic count, nuclear pleomorphism capillary proliferation, and divergent histology) were used. We assigned tumors as grade 4 when high-level mitosis, with more than 10 mitoses per 10 high-power fields, and any of the following were present: divergent histology, nuclear pleomorphism of more than 20-fold, and more than 20 capillary lumens per papillary core. The other two upgrading schemes (grade 1 to grade 2, and grade 2 to grade 3) were similar, but slightly different from this scheme.

Capillary proliferation has been evaluated in terms of the number of capillary lumina per papillary core that was cross-sectioned, and microvessel density (MVD) has been studied as a prognostic factor in many solid tumors [28, 29]. MVD could not be determined in this study, because endothelial marker immunostaining was performed in a limited number of cases of Ta tumors. However, we evaluated the light microscopic neovascularization by counting the number of capillary lumina in the most vasoproliferative papillary core. The presence of more than 20 capillary lumina was correlated with a worse clinical outcome.

A divergent histology was defined as identifiable histological features differing from the usual urothelial carcinoma. A significant number of high-grade urothelial carcinomas demonstrated glandular or squamous differentiation. In this study, tumors with divergent histology showed a worse clinical outcome than those that were pure urothelial carcinomas. The divergent histology could represent a dedifferentiation with molecular events resulting in a gain of function. Cheng et al. classified tumors with divergent differentiation, such as the nested variant, micropapillary variant, plasmacytoid variant, sarcomatoid carcinoma, small-cell carcinoma, large-cell undifferentiated carcinoma, and pleomorphic giant cell carcinoma, as grade 4 tumors [3]. In our univariate analyses, divergent histology was associated with progression of PUC-T1, but it showed less statistical significance in PUC-Ta, with a *P*-value of 0.2. Most histological parameters played no significant roles in the clinical outcome of PUC-T1, except for divergent histology. This indicated that the presence of divergent differentiation should be considered, particularly in invasive carcinoma. The reason for the reduced significance of divergent histology in the prediction of clinical outcome in PUC-Ta may be related to the low frequency of Ta stage tumors. Aggressive tumors with a divergent histology were more apparent in the invasive stage (T1) and were not usually detected at the Ta stage. Thus, we included divergent histology as one of adverse histological parameters for upgrading. Large cohort studies of PUC-Ta with a significant number of tumors with divergent histologic differentiation may be needed to verify whether this parameter has a clear biological impact.

Necrosis or apoptosis may be detected easily in a low-power view, but differentiation between these two features was not easy. In addition, degeneration of the papillary cores with dystrophic calcification could be confused with necrosis.

The newly proposed grading system designed here was compared with previous grading systems. Even though the difference in the AUCs between them was not statistically significant, the AUCs of the new grading system were larger than those of the previous grading systems for the prediction of PUC-Ta recurrence. The former AUC was more than 0.7 (*p* < 0.05), but that of the latter was less than 0.7. In addition, our proposed grading system was focused on only few, but the most powerful histological parameters, which are not descriptive or subjective, are rather quantifiable and are more reproducible, for practical use. Therefore, our system may be a better option to use as a grading system if it has a similar power for the prediction of the clinical outcome of PUC-Ta.

Because this study was not prospectively designed, with a controlled biopsy protocol and treatment, the treatment factors cannot be considered in the clinical outcome. Resection only vs. intravesical chemo/Bacille de Calmette-Guérin (BCG) treatment cannot be separately reviewed among the same grade and stage tumors. However, this study is valuable because it provided a comprehensive analysis of all histological parameters, including mitotic level and count, through a nationwide multicenter study, involving experienced uropathologists.

Although diagnostic improvement should be verified by means of a kappa value, we were unable to do so in this study. In the near future, we will collect “gray zone” tumors, with divergent designations by pathologists, and apply the new grading system to determine whether it allows improved diagnosis.

## Conclusion

The mitotic level based on the initial biopsy appears to be an independent predictor of the PUCa-Ta outcome. This finding could potentially help distinguish between low and high grade tumors in borderline lesions. Therefore, this result may help in selecting patients for a therapeutic strategy, based on the initial biopsy of NMIPUC of the bladder.

## Additional file

Additional file 1: Table S1. Univariate analysis of parameters influencing recurrence at PUC-T1 **Table S2**. Univariate analysis of parameters influencing progression at PUC-T1 **Figure S1**. Comparison of AUC predicting recurrence and progression between previous (G1, G2, G3) and our grading systems (A, B, and C). (DOCX 174 kb)

## Abbreviations

AUC: Area under the curve
CIS: Carcinoma in situ
EAU: European Association of Urology
H&E: Hematoxylin and eosin
HPF: High power field
ISUP: International Society of Urologic Pathology
LMP/LG/HG: Papillary urothelial neoplasm low malignant potential/low grade/high grade
MVD: Microvessel density
NE: No event R; recurrence, P; progression
NMIPUC: Non-muscle invasive papillary urothelial carcinoma
PHH3: Phospohistamine H3
PUC: Papillary urothelial carcinoma
ROC: Receiver operating characteristics
T1: Invasion of the lamina propria/submucosa
Ta: Non-invasive intraepithelial
WHO: World Health Organization

## References

[1] Sylvester RJ, van der Meijden AP, Oosterlinck W, Witjes JA, Bouffioux C, Denis L (2006). Predicting recurrence and progression in individual patients with stage Ta T1 bladder cancer using EORTC risk tables: a combined analysis of 2596 patients from seven EORTC trials. Eur Urol.

[2] Babjuk M, Böhle A, Burger M, Capoun O, Cohen D, Compérat EM (2017). EAU guidelines on non-muscle-invasive urothelial carcinoma of the bladder: update 2016. Eur Urol.

[3] Cheng L, MacLennan GT, Lopez-Beltran A (2012). Histologic grading of urothelial carcinoma: a reappraisal. Hum Pathol.

[4] Cheng L, Lopez-Beltran A, Bostwick DG (2012). Grading of bladder cancer. Bladder pathology.

[5] MacLennan GT, Kirkali Z, Cheng L (2007). Histologic grading of noninvasive papillary urothelial Neoplasms. Eur Urol.

[6] Shim JW, Cho KS, Choi YD, Park YW, Lee DW, Han WS (2008). Diagnostic algorithm for papillary urothelial tumors in the urinary bladder. Virchows Arch.

[7] Van Rhijn BWG, Vis AN, Van der Kwast TH, Kirkel WJ, Radvanyi F, Ooms EC (2003). Molecular grading of Urothelial cell carcinoma with fibroblast growth factor receptor 3 and MIB-1 is superior to pathologic grade for the prediction of clinical outcome. J Clin Oncol.

[8] Birkhahn M, Mitra AP, Williams AJ, Lam G, Ye W, Datar RH (2010). Predicting recurrence and progression of noninvasive papillary bladder cancer at initial presentation based on quantitative gene expression profiles. Eur Urol.

[9] Aron M, Luthringer DJ, McKenney JK, Hansel DE, Westfall DE, Parakh R (2013). Utility of a triple antibody cocktail intraurothelial neoplasm-3 (IUN-3-CK20/CD44s/p53) and α-methylacyl-CoA racemase (AMACR) in the distinction of urothelial carcinoma in situ (CIS) and reactive urothelial atypia. Am J Surg Pathol.

[10] Raspollini MR, Minervini A, Lapini A, Lanzi F, Rotellini M, Baroni G (2013). A proposed score for assessing progression in pT1 high-grade Urothelial carcinoma of the bladder. Appl Immunohistochem Mol Morphol.

[11] Rajcani J, Kajo K, Adamkov M, Moravekova E, Lauko L, Felcanova D (2013). Immunohistochemical characterization of urothelial carcinoma. Brastisl Lek Listy.

[12] Amin MB, Trpkov K, Lopez-Beltran A, Grignon D (2014). Members of the ISUP Immunohistochemistry in diagnostic Urologic pathology group. Best practices recommendations in the application of immunohistochemistry in the bladder lesions. Report from the International Society of Urologic Pathology consensus conference. Am J Surg Pathol.

[13] Tuna B, Yörükoglu K, Duzcan E, Sen S, Nese N, Sarsık B (2011). Histologic grading of urothelial; papillary neoplasms: impact of combined grading (two-numbered grading system) on reproducibility. Virchows Arch.

[14] Gonul II, Poyraz A, Unsal C, Acar C, Alkibay T (2007). Comparison of 1998 WHO/ISUP and 1973 WHO classifications for interobserver reliability in grading of papillary urothelial neoplasm of the bladder. Pathological evaluation of 258 cases. Urol Int.

[15] Bol MG, Baak JP, Buhr-Wildhagen S, Kruse AJ, Kjellevold KH, Janssen EA (2003). Reproducibility and prognostic variability of grade and lamina propria invasion in stages Ta, T1 urothelial carcinoma of the bladder. J Urol.

[16] Reuter VE, Comperat E, Algava F, Moch H, Humphrey PA, Ulbright TM, Reuter VE (2016). Non-invasive urothelial lesions. WHO classification of tumors of the urinary system and male genital organs.

[17] Bol MG, Baek JP, de Bruin PC, Rep S, Marx W, Bos S (2001). Improved objectivity of grading of T_A,1_ transitional cell carcinomas of the urinary bladder by quantitative nuclear and proliferation related factors. J Clin Pathol.

[18] Oosterhuis JW, Schapers RF, Janssen-Heijien ML, Smeets AW, Pauwels RP (2000). MIB-1 as a proliferative marker in transitional cell carcinoma of the bladder. Clinical significance and comparison with other prognostic factors. Cancer.

[19] Malpica A, Deavers MT, Lu K, Bodurka DC, Atkinson EN, Gershenson DM (2004). Grading ovarian serous carcinoma using a two-tier system. Am J Surg Pathol.

[20] Robbins P, Pinder S, de Klerk N, Dawkins H, Harvey J, Sterrett G (1995). Histological grading of breast carcinomas: a study of interobserver agreement. Hum Pathol.

[21] Mangurud OM, Gudlaugsson E, Skaland I, Tasdemir I, Dalen I, van Diermen B (2014). Prognostic comparison of proliferation makers and World Health Organization 1973/2004 grade in urothelial carcinoma of the urinary bladder. Hum Pathol.

[22] Watts KE, Montironi R, Mazzucchelli R, van der Kwast T, Osunkoya AO, Stephenson AJ (2012). Clinicopathologic characteristics of 23 cases of invasive low grade papillary urothelial carcinoma. Urologia.

[23] Goyal S, Singl UR, Sharma S, Kaur N (2014). Correlation of mitotic indices, AgNor count, Ki-67 and Bcl-2 with grade and stage in papillary urothelial bladder cancer. Urol J.

[24] Pich A, Chiusa L, Formiconi A, Galliano D, Bortolin P, Comino A (2002). Proliferative activity is the most significant predictor of recurrence in noninvasive papillary urothelial neoplasms of low malignant potential and grade 1 papillary carcinomas of the bladder. Cancer.

[25] Akkalp AK, OPnur O, Tetikkurt US, Tolga D, Özsoy S, Müslümanoğlu AY (2016). Prognostic significance of mitotic activity in noninvasive, low grade, papillary urothelial carcinoma. Anal Quant Cytopathol Histopathol.

[26] Solomides CC, Birbe R, Nicolau N, Bagley D, Bibbo M (2012). Does mitosis- specific marker phophohistone H3 help the grading of upper tract urothelial carcinomas in cell blocks?. Acta Cytol.

[27] Van Rhijn WG, Musquera M, Liu L, Vis AN, Zuiverloon TC, van Leenders GJ (2014). Molecular and clinical support for a four-tiered grading system for bladder cancer based on the WHO 1973 and 2004 classifications. Mod Pathol.

[28] Santos L, Costa C, Pereira S, Koch M, Amaro T, Cardoso F (2003). Neovascularization is a prognostic factor of early recurrence in T1/G2 urothelial bladder tumors. Ann Oncol.

[29] Jang TJ, Kim SW, Lee KS (2012). The expression of pigment epithelium-derived factor in bladder transitional cell carcinoma. Korean J Pathol.

