# Supplementary material for: Level of mitoses in non-muscle invasive papillary urothelial carcinomas (pTa and pT1) at initial bladder biopsy is a simple and powerful predictor of clinical outcome: a multi-center study in South Korea
Source: Diagn Pathol. 2017 Jul 24;12:54. doi: 10.1186/s13000-017-0639-y (PMC5525253; doi:10.1186/s13000-017-0639-y)

**Table S1. Univariate analysis of parameters influencing recurrence at PUC-T1**

| **Variable** | **Category** | **Odds ratio** | **95% Confidence Interval** | **P-value** |
| --- | --- | --- | --- | --- |
| **Age** |  | 1.01 | 0.97-1.05 | 0.6023 |
| **Sex** | **man vs. woman** | 1.05 | 0.32-3.27 | 0.9317 |
| **No of tumor** |  | 0.69 | 0.28-1.69 | 0.4169 |
| **Size of tumor** |  | 1.63 | 0.66-4.16 | 0.2988 |
| **Papillary fusion** | **2 vs 1** | 0.59 | 0.18-1.89 | 0.3775 |
|  | **3 vs 1** | 0.81 | 0.26-2.40 | 0.7022 |
| **Umbrella cell** | **2 vs. 1** | 0.00 | NA | 0.9921 |
|  | **3 vs. 1** | 0.00 | NA | 0.9923 |
| **Discohesiveness** | **2 vs. 1** | 0.55 | 0.18-1.62 | 0.2810 |
|  | **3 vs. 1** | 1.42 | 0.48-4.37 | 0.5253 |
| **Cell density** | **2 vs. 1** | 3.67 | 0.57-27.1 | 0.1765 |
|  | **3 vs. 1** | 2.00 | 0.41-10.92 | 0.3909 |
| **Nuclear pleomorphism** | **2 vs. 1** | 3.00 | 0.42-26.53 | 0.2769 |
|  | **3 vs. 1** | 3.56 | 0.5-31.28 | 0.2064 |
|  | **4 vs. 1** | 1.50 | 0.21-12.86 | 0.6832 |
| **Multinucleated giant cell** | **1 vs. 0** | 1.47 | 0.46-4.58 | 0.5035 |
| **Loss of polarity** | **2 vs. 1** |  |  |  |
|  | **3 vs. 1** |  |  |  |
| **Hyperchromasia** | **2 vs. 1** | 1.03 | 0.28-3.71 | 0.9636 |
|  | **3 vs. 1** | 1.07 | 0.3-3.58 | 0.9173 |
| **Nuclear groove** | **2 vs. 1** | 4.20 | 0.7-34.18 | 0.1298 |
|  | **3 vs. 1** | 3.00 | 0.53-23.31 | 0.2314 |
| **Prominent nucleoli** | **2 vs. 1** | 0.81 | 0.32-2.07 | 0.6606 |
|  | **3 vs. 1** | 0.88 | 0.13-7.18 | 0.8937 |
| **Whorling pattern** | **1 vs. 0** | 0.36 | 0.14-0.92 | 0.0341 |
| **Necrosis** | **2 vs 1** | 4.89 | 1.00-29.59 | 0.0607 |
|  | **3 vs 1** | 1.89 | 0.52-7.14 | 0.3342 |
|  | **4 vs 1** | 2.37 | 0.63-9.43 | 0.2058 |
| **Divergent histology** |  | 0.83 | 0.26-2.76 | 0.7470 |
| **Mitotic count** |  | 1.01 | 0.99-1.04 | 0.1927 |
| **Mitotic count** | **2 vs. 1** | 20868481.10 | NA | 0.9921 |
|  | **3 vs. 1** | 25042177.32 | NA | 0.9920 |
|  | **4 vs. 1** | 28007698.32 | NA | 0.9919 |
| **Mitosis level** | **2 vs. 1** |  |  |  |
|  | **3 vs. 1** |  |  |  |
| **Apoptosis** | **2 vs 1** | 1.48 | 0.53-4.19 | 0.4591 |
|  | **3 vs 1** | 0.78 | 0.24-2.53 | 0.6827 |
| **Capillary proliferation** |  | **1.03** | **0.98-1.08** | **0.2157** |
| **Table S2. Univariate analysis of parameters influencing progression at PUC-T1** |  |  |  |  |
| **Variable** | **Category** | **Odds ratio** | **95% Confidence Interval** | **P-value** |
| **Age** |  | 1.02 | 0.99-1.06 | 0.1913 |
| **Sex** | **man vs. woman** | 0.58 | 0.23-1.49 | 0.2487 |
| **No of tumor** |  | 0.50 | 0.21-1.15 | 0.1118 |
| **Size of tumor** |  | 1.13 | 0.51-2.49 | 0.7629 |
| **Papillary fusion** | **2 vs 1** | 0.58 | 0.19-1.71 | 0.3326 |
|  | **3 vs 1** | 1.00 | 0.41-2.51 | 1.0000 |
| **Umbrella cell** | **2 vs. 1** | 1.04 | 0.23-5.63 | 0.9571 |
|  | **3 vs. 1** | 2.30 | 0.66-10.74 | 0.2274 |
| **Discohesiveness** | **2 vs. 1** | 0.87 | 0.33-2.24 | 0.7742 |
|  | **3 vs. 1** | 0.74 | 0.28-1.88 | 0.5322 |
| **Cell density** | **2 vs. 1** | 0.54 | 0.13-2.25 | 0.3987 |
|  | **3 vs. 1** | 0.45 | 0.13-1.52 | 0.1857 |
| **Nuclear pleomorphism** | **2 vs. 1** | 0.47 | 0.1-2.26 | 0.3291 |
|  | **3 vs. 1** | 0.79 | 0.18-3.57 | 0.7458 |
|  | **4 vs. 1** | 0.29 | 0.06-1.46 | 0.1246 |
| **Multinucleated giant cell** | **1 vs. 0** | 1.39 | 0.49-4.57 | 0.5583 |
| **Loss of polarity** | **2 vs. 1** | 0.00 | NA | 0.9871 |
|  | **3 vs. 1** | 0.00 | NA | 0.9869 |
| **Hyperchromasia** | **2 vs. 1** | 1.18 | 0.35-4.32 | 0.7933 |
|  | **3 vs. 1** | 1.54 | 0.51-5.28 | 0.4616 |
| **Nuclear groove** | **2 vs. 1** | 2.71 | 0.41-53.72 | 0.3765 |
|  | **3 vs. 1** | 2.80 | 0.44-54.65 | 0.3545 |
| **Prominent nucleoli** | **2 vs. 1** | 1.76 | 0.79-3.99 | 0.1684 |
|  | **3 vs. 1** | 0.57 | 0.03-3.93 | 0.6253 |
| **Whorling pattern** | **1 vs. 0** | 0.49 | 0.18-1.22 | 0.1410 |
| **Necrosis** | **2 vs 1** | 0.71 | 0.17-2.78 | 0.6292 |
|  | **3 vs 1** | 0.69 | 0.22-2.25 | 0.5292 |
|  | **4 vs 1** | 1.12 | 0.37-3.55 | 0.8426 |
| **Divergent histology** |  | 2.59 | 1.24-5.82 | 0.0154 |
| **Mitotic count** |  | 1.00 | 0.99-1.02 | 0.7321 |
| **Mitotic count** | **2 vs. 1** | 0.07 | 0-1.06 | 0.0667 |
|  | **3 vs. 1** | 0.54 | 0.05-5.29 | 0.5751 |
|  | **4 vs. 1** | 0.49 | 0.06-4.27 | 0.4885 |
| **Mitosis level** | **2 vs. 1** | 0.00 | NA | 0.9905 |
|  | **3 vs. 1** | 0.00 | NA | 0.9910 |
| **Apoptosis** | **2 vs 1** | 1.60 | 0.6-4.47 | 0.3530 |
|  | **3 vs 1** | 2.48 | 0.88-7.37 | 0.0911 |
| **Capillary proliferation** |  | 1.01 | 0.98-1.04 | 0.5778 |

NA: not available

**Figure S1. Comparison of AUC predicting recurrence and progression between previous (G1, G2, G3) and our grading systems (A, B, and C)**


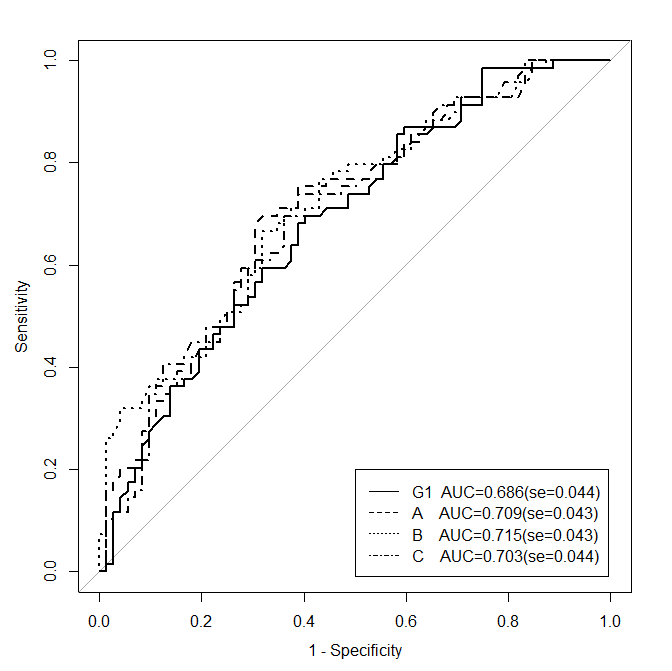

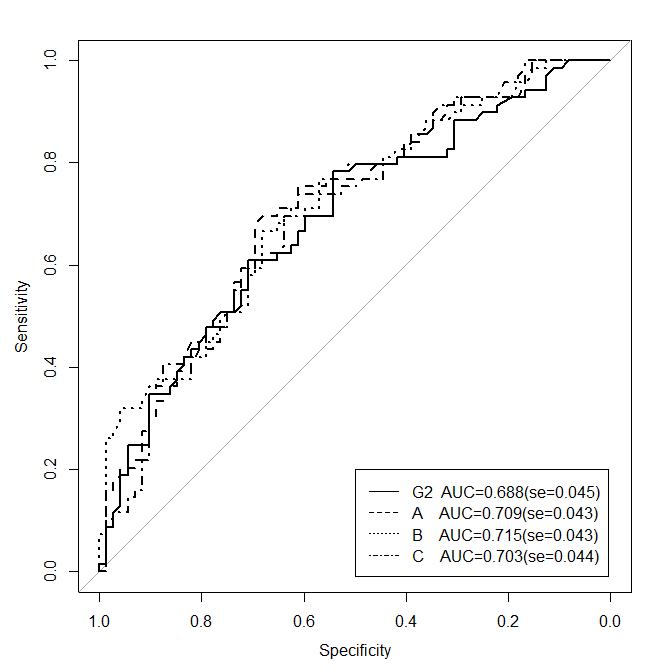

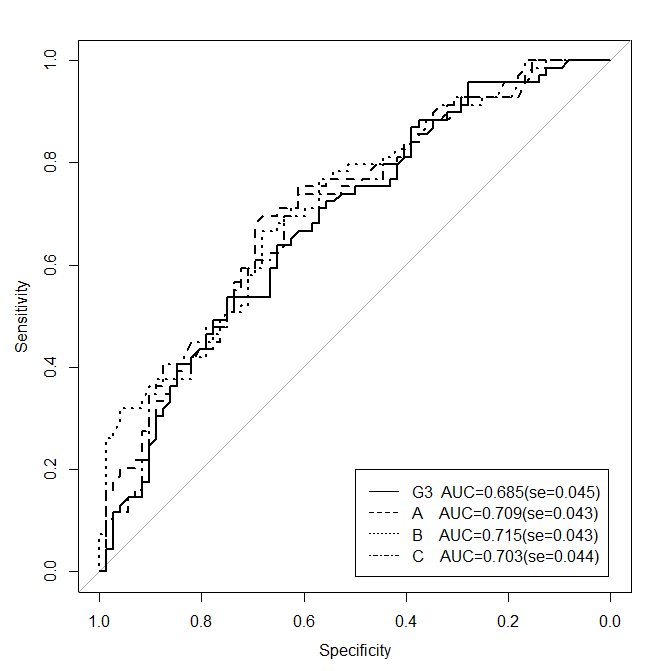

Supplement: Additional file 1: Table S1. — Univariate analysis of parameters influencing recurrence at PUC-T1 Table S2. Univariate analysis of parameters influencing progression at PUC-T1 Figure S1. Comparison of AUC predicting recurrence and progression between previous (G1, G2, G3) and our grading systems (A, B, and C). (DOCX 174 kb) [file 13000_2017_639_MOESM1_ESM.docx]
